# Rigid, bivalent CTLA-4 binding to CD80 is required to disrupt the *cis* CD80/PD-L1 interaction

**DOI:** 10.1016/j.celrep.2024.114768

**Published:** 2024-09-14

**Authors:** Maximillian A. Robinson, Alan Kennedy, Carolina T. Orozco, Hung-Chang Chen, Erin Waters, Dalisay Giovacchini, Kay Yeung, Lily Filer, Claudia Hinze, Christopher Lloyd, Simon J. Dovedi, and David M. Sansom

**Affiliations:** 1 Institute of Immunity and Transplantation, Pears Building, Rowland Hill St, London NW3 2PP, UK; 2 Biologics Engineering, R&D, AstraZeneca, 1, Francis Crick Avenue Cambridge CB2 0AA, UK; 3 Early Oncology ICC, R&D, AstraZeneca, 1, Francis Crick Avenue, Cambridge CB2 0AA, UK

## Abstract

The CTLA-4 and PD-1 checkpoints control immune responses and are key targets in immunotherapy. Both pathways are connected via a *cis* interaction between CD80 and PD-L1, the ligands for CTLA-4 and PD-1, respectively. This *cis* interaction prevents PD-1-PD-L1 binding but is reversed by CTLA-4 *trans*-endocytosis of CD80. However, how CTLA-4 selectively removes CD80, but not PD-L1, is unclear. Here, we show CTLA-4-CD80 interactions are unimpeded by PD-L1 and that CTLA-4 binding with CD80 does not displace PD-L1 *per se*. Rather, both rigidity and bivalency of CTLA-4 molecules are required to orientate CD80 such that PD-L1 interactions are no longer permissible. Moreover, soluble CTLA-4 released PD-L1 only at specific expression levels of CD80 and PD-L1, whereas CTLA-4 *trans*-endocytosis released PD-L1 in all conditions. These data show that PD-L1 release from CD80 is driven by orientation and bivalent cross-linking of membrane proteins and that *trans*-endocytosis of CD80 efficiently promotes PD-L1 availability.

Graphical abstract

## Graphical abstract

**Graphical abstract:**
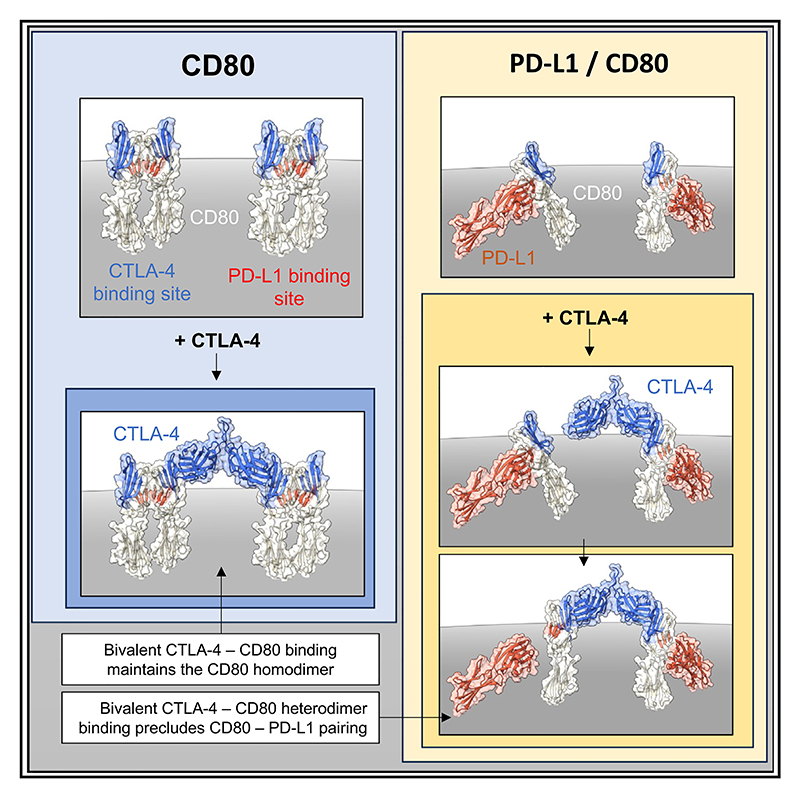

## Introduction

CTLA-4 is a critical immune checkpoint in the attenuation of T cell responses. Homozygous CTLA-4 deletion in mice leads to a fatal, lympho-proliferative phenotype, a result of excessive T cell activation due to a deficiency in the regulatory T cell compartment.^1,2^ Homozygous CTLA-4 mutations in humans have not been reported and are assumed fatal; however, patients with heterozygous mutations in the *CTLA4* gene often present with severe immune dysregulation and autoimmunity.^3^

CTLA-4 is an endocytic receptor that functions as the primary antagonist of the CD28 co-stimulatory pathway.^2^ Both CD28 and CTLA-4 share two ligands, CD80 and CD86; however, CTLA-4 binds both with a greater affinity than CD28.^4^ We have shown that CTLA-4 physically depletes CD80 and CD86 in a process called *trans*-endocytosis.^5^ By binding and removing CD80 and CD86 from opposing cells, CTLA-4 can regulate the amount of co-stimulatory ligand available for CD28-driven T cell activation. Moreover, the identity of the ligand bound also impacts CTLA-4 fate and the levels of functional CTLA-4.^6^ These observations highlight the cell-extrinsic nature of CTLA-4 function, in keeping with experimental results from mouse studies and the observed function of regulatory T cells.^7,8^

Research into CTLA-4 has yielded several successful clinically approved therapies. Abatacept (CTLA-4 Ig), a CTLA-4-immunoglobulin (Ig) fusion protein, functions by binding free CD80 and CD86, blocking ligation of CD28, and therefore attenuating T cell responses. Abatacept was first approved for patients with rheumatoid arthritis but has since been indicated for the treatment of psoriatic arthritis, graft vs. host disease (GVHD), and, more recently, CTLA-4 deficiency syndromes.^9–13^ In contrast, immunotherapies that block CTLA-4 have revolutionized the treatment of cancer and function by increasing CD28 activity, thereby potentiating the T cell response.^14^ However, anti-CTLA-4 therapies have high levels of immune-related adverse events, which sub-optimally limit pathway inhibition.^15^ Moreover, targeting the PD-1 receptor, another cell-surface protein involved in T cell inhibition, has emerged for many cancer indications. Combination therapies blocking both the CTLA-4 and PD-1 receptors demonstrate increased responses as compared to either CTLA-4 or PD-1 blockade alone.^16,17^

Despite initial concepts that CTLA-4 and PD-1 represent discrete pathways, recently, it has emerged that there is a significant molecular overlap between the CTLA-4 and PD-1 pathways. Butte et al. first demonstrated that CD80 and PD-L1 interact with a low micromolar affinity^18^; subsequently, CD80 and PD-L1 were shown to predominately interact in *cis*, when both ligands are co-expressed on the same cell membrane.^19^ Moreover, the interaction between CD80 and PD-L1 precludes PD-1 binding, thus preventing PD-1-mediated inhibition.^19,20^ Mutational analysis suggests that the CD80 and PD-1 binding sites on PD-L1 are overlapping, with further structural data confirming that residues on the PD-L1 protein that mediate PD-1 binding are shared with CD80.^21^ Thus, it appears likely that CD80 inhibits PD-1/PD-L1 interactions by steric obstruction of the PD-1 binding site.

In contrast, CD80 remains available for CTLA-4 binding despite being in complex with PD-L1.^22^ *In vitro* cellular assays demonstrated that *trans*-endocytosis of CD80 by CTLA-4 was unimpeded by the CD80 interaction with PD-L1 and that CD80 depletion was remarkably specific, as PD-L1 was retained at the cell membrane despite CD80 removal. Thus, following depletion of CD80 by *trans*-endocytosis, PD-L1/PD-1 interactions are rescued, and PD-1-mediated inhibition is restored. In principle, this connects CTLA-4 function with the promotion of PD-1 inhibition. Additional reports indicated that soluble CTLA-4 variants (CTLA-4 Ig) and cell-expressed CTLA-4 mutants that are unable to perform *trans*-endocytosis were also capable of releasing PD-L1, features we did not initially observe in our system.^21,23^This raises several questions about how *trans*-endocytosis of CD80 occurs without its heterodimeric partner PD-L1, why the impact of soluble CTLA-4 is variable, and how CTLA-4 can disrupt CD80/PD-L1 interactions.

Herein, we explored the mechanism by which CTLA-4 can selectively deplete CD80 but not PD-L1. We observed that soluble CTLA-4 Ig binding to cells expressing both CD80 and PD-L1 only rescues PD-1 binding when both ligands are expressed at similar levels but not when CD80 was in excess. In addition, we generated a series of soluble CTLA-4 variants to understand why PD-L1 release was so dependent on ligand expression levels. Using both monovalent CTLA-4 constructs and flexible, bivalent CTLA-4 constructs, we demonstrate that neither could cause dissociation of the CD80/PD-L1 heterodimer. Therefore, the release of PD-L1 is not achieved by simple CTLA-4 binding to CD80/PD-L1 heterodimers *per se* but requires both bivalent and rigid binding of the wild-type (WT) CTLA-4 protein with CD80 to release PD-L1. Finally, we compared the ability of soluble CTLA-4 with cell-expressed WT CTLA-4 to release PD-L1 and show that, in contrast to soluble CTLA-4, only *trans*-endocytosis of CD80 could effectively release PD-L1, regardless of CD80 and PD-L1 expression levels.

## Results

### CTLA-4 Ig disrupts CD80/PD-L1 interactions only at defined ligand levels

**Figure 1:**
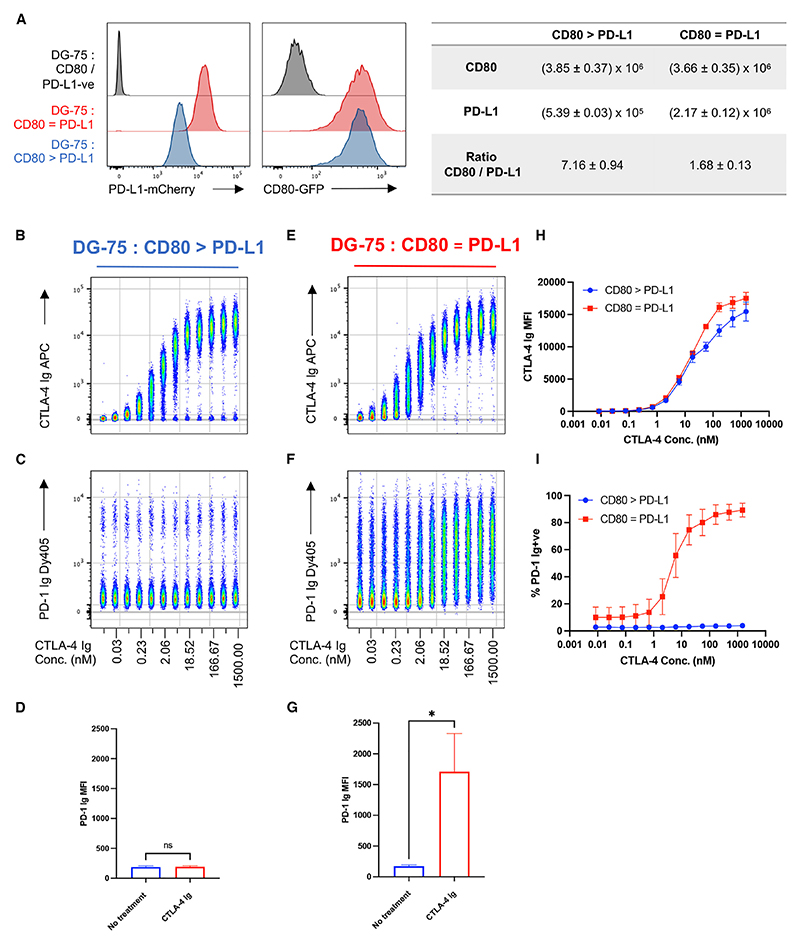
CTLA-4 Ig only disrupts CD80/PD-L1 interactions at defined ligand levels (A) Representative histograms showing PD-L1-mCherry and CD80-GFP levels on two different DG-75 cell lines. The right-hand table details ligand numbers and molecular ratio of CD80 and PD-L1 on either cell line. (B) Concatenated flow cytometry plot of a 12-point serial dilution of CTLA-4 Ig-APC, starting at 1,500 nM, on DG-75:CD80 > PD-L1. (C) PD-1 Ig-Dy405 detection following CTLA-4 Ig treatment. (D) MFI of PD-1 Ig binding to DG-75:CD80 > PD-L1 ± 1,500 nM of CTLA-4 Ig. (E–G) As in (B)–(D) but performed on DG-75:CD80 = PD-L1. (E) Graphical comparison between DG-75:CD80 = PD-L1 and DG-75:CD80 > PD-L1 of CTLA-4 Ig-APC binding at indicated concentrations. (F) Graphical comparison between DG-75:CD80 = PD-L1 and DG-75:CD80 > PD-L1 of PD-1 Ig-positive cells after incubation with indicated concentrations of CTLA-4 Ig-APC. Data are representative of three independent experiments showing mean ± SD. *p ≤ 0.05, ns, not significant: paired t test (D and G).

To study the interaction between CD80 and PD-L1, we transduced GFP-tagged CD80 and mCherry-tagged PD-L1 into a DG-75 B cell line. We sorted cell populations with varying levels of ligand and determined the molecular ratio of CD80 to PD-L1 in each independent cell line (Figure 1A) based on ligand quantitation we performed previously.^22^

We then titrated an APC-labeled CTLA-4 Ig fusion protein (CTLA-4 Ig, referred to clinically as abatacept) on a population of DG-75 cells expressing an excess of CD80 as compared to PD-L1. As observed previously,^22^ CTLA-4 Ig binding to CD80 was unimpeded by PD-L1 co-expression (Figure 1B). Next, we used a soluble PD-1 Ig fusion protein (PD-1 Ig) to determine the availability of PD-L1 for PD-1 binding. We observed that when CD80 levels were in excess of PD-L1, PD-1 Ig binding was abrogated and that this was unaffected by the presence of CTLA-4 Ig (Figure 1C). Even at the highest dose of CTLA-4 Ig, we failed to see a shift in PD-1 Ig binding (Figure 1D). Therefore, CTLA-4 binding to CD80 did not promote PD-L1 release *per se*, and a tripartite complex between CD80, PD-L1, and CTLA-4 was permitted.

We then repeated the same assay on DG-75 cells expressing approximately equal levels of PD-L1 as compared to CD80 (DG-75:CD80 = PD-L1). These cells showed a low level of baseline PD-1 Ig staining, representing some free PD-L1; however, this was clearly enhanced at increasing concentrations of CTLA-4 Ig (Figures 1E and 1F). Moreover, at the highest CTLA-4 Ig doses, we observed a significant increase in PD-1 Ig binding (Figure 1G). Importantly, the levels of CTLA-4 Ig binding were comparable between cell lines (Figure 1H), yet a concomitant increase in PD-1 Ig binding was only observed on the DG-75:CD80 = PD-L1 population (Figure 1I). Thus, we concluded that soluble CTLA-4 can enhance PD-L1/PD-1 binding, although only at favorable ratios of CD80 to PD-L1 expression.

CD28 shares significant similarities with CTLA-4: both are covalent homodimers and share the MYPPPY motif, essential for CD80 binding.^24^ We therefore tested the impact of CD28 binding on PD-L1 release. We produced a soluble CD28 Ig fusion protein (CD28 Ig) and, using biolayer interferometry (BLI), confirmed that immobilized CD28 Ig bound monomeric His-tagged CD80 (Figure S1A) with a weaker affinity (CD28/CD80 K_D_ = (4.27 ± 0.46) × 10^−5^ M) than CTLA-4, as expected.^25^ Furthermore, when titrated onto DG-75 cells expressing equal levels of CD80 and PD-L1, CD28 Ig binding to CD80 was “right-shifted” as compared to CTLA-4 Ig, reflecting its lower affinity (Figure S1B). Intriguingly, despite CD28 sharing the same binding site as CTLA-4, we failed to observe any release of PD-L1, as measured by PD-1 Ig binding (Figure S1C). Considering that the binding site on CD80 is shared between CTLA-4 and CD28, these results suggest that CTLA-4 binding to CD80 is uniquely capable of releasing PD-L1.

### CTLA-4 monovalent binding fails to disrupt CD80/PD-L1 interactions

Despite CD28 and CD28 Ig existing as covalent homodimers, biophysical analysis and structural data suggest that CD28/CD80 interactions are primarily monovalent, due to the angle of ligand binding preventing both CD28 sites from being engaged simultaneously.^4,26^ In contrast, CTLA-4 can interact with CD80 bivalently.^27^ We therefore considered the hypothesis that bivalent binding may be required to drive the observed dissociation of CD80 from PD-L1 following CTLA-4 binding.

**Figure 2:**
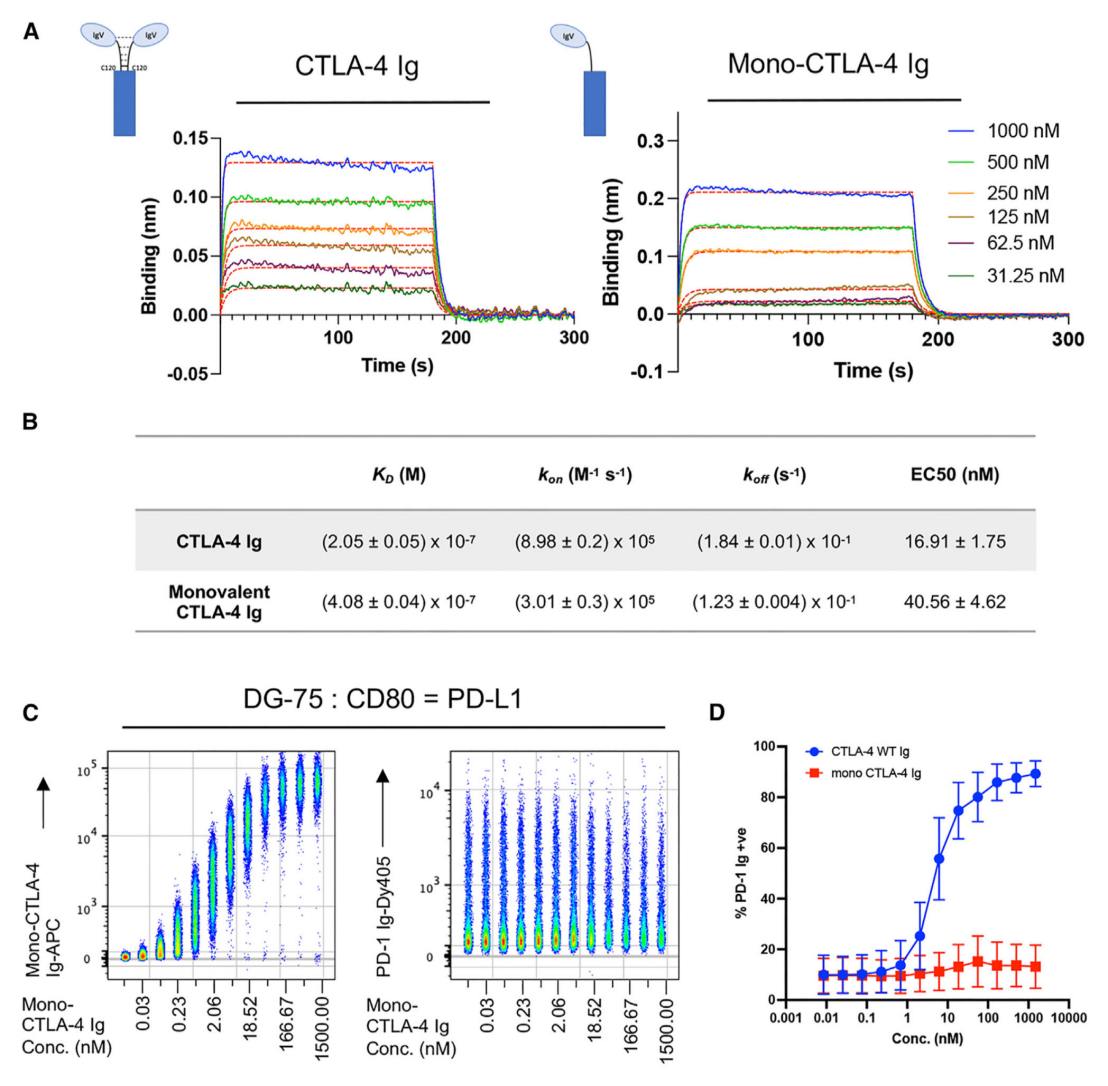
CTLA-4 monovalent binding fails to disrupt the CD80/PD-L1 interaction (A) BLI binding curves showing the association and dissociation of 1,000, 500, 250, 125, 62.5, and 31.3 nM of CD80-His to CTLA-4 Ig (left) and mono-CTLA-4 Ig (right). Red lines show best fit to a 1:1 binding model. (B) Kinetic parameters (K_D_, *k*_*on*_, *k*_*off*_) obtained from the best global fit of the association/dissociation data to a 1:1 binding model. The errors given are fitting errors from the global fitting. (C) Concatenated flow cytometry plot of a 12-point serial dilution of mono-CTLA-4 Ig-APC, starting at 1,500 nM, on DG-75:CD80 = PD-L1, followed by PD-1 Ig-Dy405 detection (right-hand side). (D) Graphical representation of PD-1 Ig-positive cells after treatment with WT CTLA-4 Ig or mono-CTLA-4 Ig at indicated concentrations. Data are representative of three independent experiments showing mean ± SD.

To test this, we generated a soluble, monovalent CTLA-4 Ig fusion protein (mono-CTLA-4 Ig) using the knobs-into-holes technology (see STAR Methods and Figure S2A for protein sequences).^28^ Affinity measurements indicated that mono-CTLA-4 Ig bound CD80 with a similar K_D_ to WT CTLA-4 Ig, suggesting that CTLA-4/CD80 affinity was similar for both constructs as calculated by both kinetic analysis (Figures 2A and 2B) and steady state (Figure S3). To confirm that our mono-CTLA-4 Ig construct bound monovalently, we performed a BLI assay using immobilized CD80 His, probing a range of concentrations of WT CTLA-4 Ig and mono-CTLA-4 Ig (Figure S4A). Even at low concentrations, WT CTLA-4 Ig dissociated poorly from CD80, a result of the enhanced avidity of the bivalent interaction (Figure S4B). In contrast, mono-CTLA-4 Ig could readily dissociate from immobilized CD80, consistent with a monovalent binding model between the ligand-receptor pair (Figure S4C). DG-75:CD80 = PD-L1 cells were then treated with a titration of mono-CTLA-4 Ig (Figure 2C). Remarkably, even at saturating doses of mono-CTLA-4 Ig, we were unable to restore PD-1 Ig binding (Figures 2C and 2D). Taken together, these results show that CTLA-4/CD80 binding alone is insufficient to release PD-L1 but that CTLA-4-mediated dissociation of the CD80/PD-L1 heterodimer is dependent upon bivalent CTLA-4 binding.

### Structural modeling of CTLA-4 interacting with the PD-L1/CD80 heterodimer

**Figure 3:**
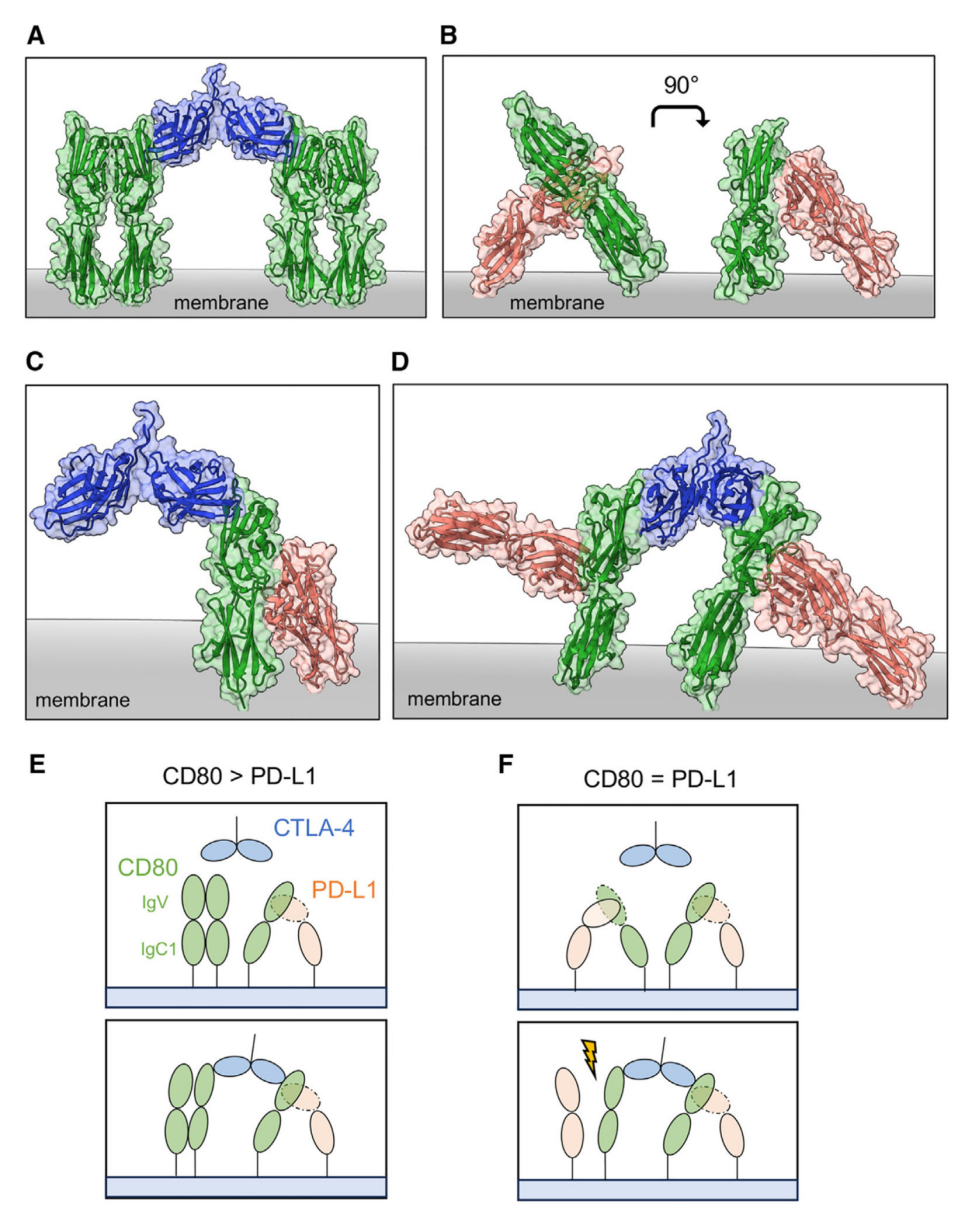
Structural models of CTLA-4 interacting with the PD-L1/CD80 heterodimer (A) Representative surface and ribbon structure of CTLA-4 (blue) in complex with two CD80 homodimers (green) (PDB: 1I8L). (B) Rotated view of the ALPN-202 CD80 vIgD/PD-L1 ECD (red) asymmetric unit aligned with WT CD80 (green) (PDB: 7TPS and 1DR9). (C) Model alignments of the CD80/PD-L1 complex interacting with CTLA-4 monovalently. (D) Bivalent CTLA-4 binding to PD-L1/CD80 heterodimers (PDB: 7TPS, 1DR9, and 1I8L); structure has been rotated to highlight the angle of CD80 relative to the membrane and displacement of the PD-L1 protein. (E and F) Schematic depicting CTLA-4 (blue) binding to CD80 (green)/PD-L1 (red) heterodimers when CD80 is expressed in excess to PD-L1 (E) and when CD80 and PD-L1 are expressed at equimolar ratios (F).

CD80 is composed of a membrane-distal Ig variable (IgV) domain and a membrane-proximal Ig constant (IgC1) domain, which forms transient, non-covalent homodimers (mediated via IgV domain interactions). These dimers interact with low affinity and a reported K_D_ of 20–50 μM.^29^ CD80 dimerization results in two monomers arrayed parallel to one another and orthogonal to the cell membrane in a conformation that is maintained when complexed with CTLA-4 (Figure 3A).^27^

Although the full crystal structure of CD80 interacting with PD-L1 is yet to be determined, a recent paper resolved the structure of a variant high-affinity CD80 IgV domain (ALPN-202 CD80 vIgD) in complex with PD-L1.^30^ We therefore aligned the structure of WT CD80 based upon the high-affinity CD80/PD-L1 crystal structure (Figure 3B). In this model, PD-L1 binds the CD80 dimer interface at an unusual angle (“lying down”) such that it would appear to prevent CD80 from existing at an orthogonal angle with the membrane. Nonetheless, the model indicates that both CTLA-4 and PD-L1 can simultaneously bind to CD80 (Figure 3C). However, by changing the angle of CD80 in the membrane, CTLA-4 interactions are then skewed when compared to CD80 homodimer binding. Furthermore, it appears unlikely that CTLA-4 can bridge two CD80/PD-L1 complexes, as both PD-L1 molecules can no longer anchor in the membrane (Figure 3D). Structural analysis therefore suggests that bivalent binding of CTLA-4 to two CD80/PD-L1 heterodimers is unlikely, offering an explanation as to why the release of PD-L1 depends on the relative levels of CD80 and PD-L1 in the membrane.

Accordingly, if CD80 levels are in excess to PD-L1, membrane CD80 will form a mixed population of transient CD80 homodimers and CD80/PD-L1 heterodimers. Thus, CTLA-4 could bivalently bind with one arm to a CD80/PD-L1 heterodimer, with the other arm binding a CD80 monomer or homodimer, thereby failing to release PD-L1 (Figure 3E). In the context of equimolar expression of CD80 and PD-L1, the majority of CD80 ligand is complexed with PD-L1. Here, bivalent binding is precluded until CD80 and PD-L1 dissociate, only then allowing CTLA-4 to bridge a PD-L1/CD80 heterodimer and a CD80 monomer (Figure 3F). Moreover, once formed, bivalent CTLA-4/CD80 complexes would likely prevent PD-L1 re-association and therefore generate detectable free PD-L1. Therefore, the only condition in which we observe significant, but ultimately partial, restoration of PD-1/PD-L1 binding by soluble CTLA-4 is under conditions where the vast majority of CD80 at the membrane is in complex with PD-L1.

### Rigid CTLA-4 dimers are required to restore PD-1 binding

The above model suggests that, in addition to bivalency, the geometry of CTLA-4 interacting with CD80 is important. This geometry is constrained by both the binding epitope and the rigidity of the CTLA-4 dimer. Indeed, the CTLA-4/CD80 interaction has been described as a rigid body since neither molecule’s conformation is changed when comparing their *apo* and complexed crystal structures.^31^ We therefore tested whether rigidity of the CTLA-4 homodimer was required to mediate PD-L1 release by producing flexible CTLA-4 molecules. We produced two CTLA-4 Ig variants: one where we substituted the native cysteine, required for the disulfide bridge, with an alanine (CTLA-4 C120A Ig) and a second variant where we removed the CTLA-4 stalk region and replaced it with a flexible GGGGS linker (CTLA-4 V114 G4S Ig) (Figure S2A).

**Figure 4:**
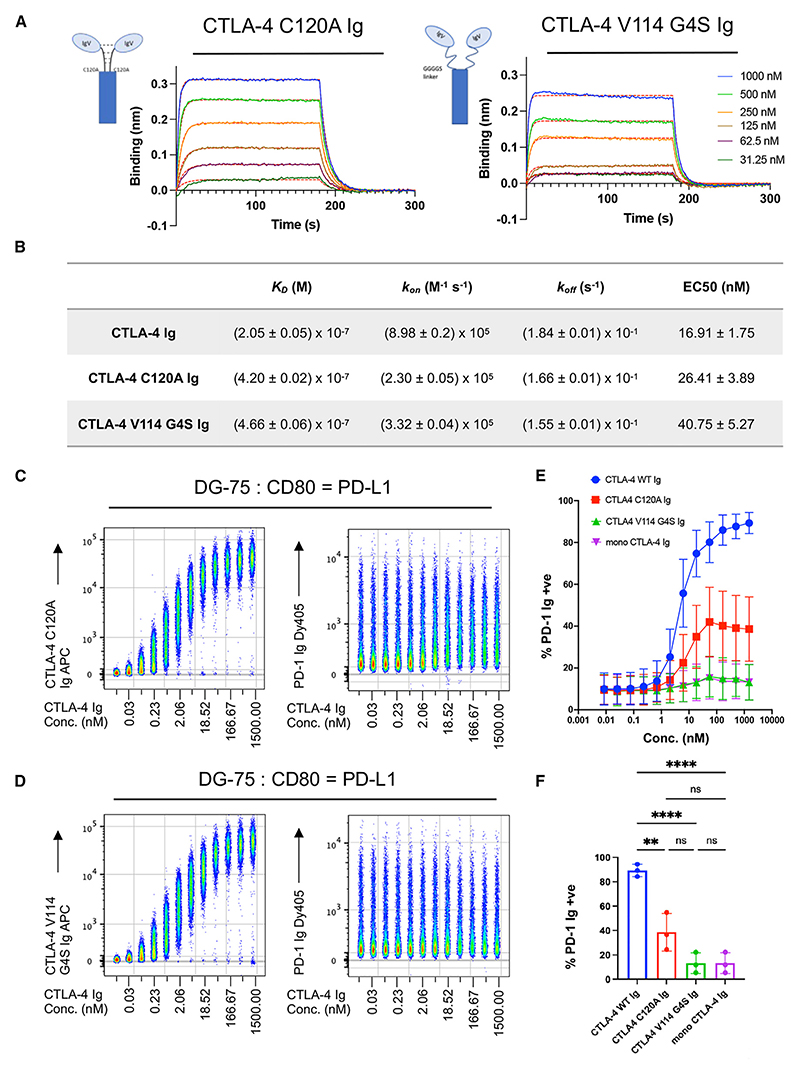
Rigid CTLA-4 binding is required to restore PD-1 binding (A) Binding curves showing the association and dissociation of 1,000, 500, 250, 125, 62.5, and 31.3 nM of CD80-His to CTLA-4 C120A Ig (left) and CTLA-4 V114 G4S Ig (right). Red lines show best fit to a 1:1 binding model. (B) Kinetic parameters (K_D_, *k*_*on*_, *k*_*off*_) obtained from the best global fit of the association/dissociation data to a 1:1 binding model. The errors given are fitting errors from the global fitting. (C and D) Concatenated flow cytometry plot of a 12-point serial dilution of CTLA-4 C120A Ig-APC (C) and CTLA-4 V114 G4S Ig-APC (D), starting at 1,500 nM, on DG-75:CD80 = PD-L1, followed by PD-1 Ig-Dy405 detection (right-hand sides). (E) Graphical representation of PD-1 Ig-positive DG-75:CD80 = PD-L1 cells, after treatment with indicated CTLA-4 Ig construct, at indicated concentrations. (F) Percentage of PD-1 Ig+ve DG-75:CD80 = PD-L1 after treatment with 1,500 nM of indicated CTLA-4 construct. Data are representative of three independent experiments showing mean ± SD. **p ≤ 0.01, ****p ≤ 0.0001, ns, not significant: one-way ANOVA with Tukey’s multiple comparisons test (F).

BLI analysis confirmed these constructs were both able to bind CD80. Immobilized CTLA-4 C120A Ig and CTLA-4 V114 G4S Ig bound soluble CD80-His with a similar affinity to WT CTLA-4 Ig (Figures 4A, 4B, S3C, and S3D); given that binding between CTLA-4 and CD80 utilizes a ligand-binding site in the IgV domain, alterations in the stalk region had little impact on binding, as expected. Using these constructs in a BLI assay with immobilized CD80-His, we observed slow dissociation (as seen with WT CTLA-4 Ig), indicating that avidity-enhanced bivalent interactions still occurred with these constructs (Figures S4D and S4E).

When these flexible, bivalent constructs were titrated onto DG-75:CD80 = PD-L1, we observed a similar EC50 to WT CTLA-4 Ig, demonstrating that our CTLA-4 Ig variants readily bound CD80 in complex with PD-L1 (Figure 4B). However, at saturating concentrations, both CTLA-4 C120A Ig and CTLA-4 V114 G4S Ig failed to restore PD-1 Ig binding to the level seen with WT CTLA-4 Ig (Figures 4C and 4D). Interestingly, the highly flexible CTLA-4 V114 G4S Ig failed to alter PD-1 Ig binding whatsoever, indicating that structural rigidity was required to dissociate the CD80/PD-L1 heterodimer. However, we observed that, at high doses, CTLA-4 C120A Ig could release low levels of PD-L1, as measured by PD-1 Ig binding, suggesting an intermediate phenotype (Figures 4E and 4F). Therefore, by comparing three novel constructs (mono-CTLA-4 Ig, CTLA-4 C120A Ig, and CTLA-4 V114 G4S Ig), we concluded that the ability to effect PD-L1 release and restore PD-1 Ig binding required rigid, bivalent CD80-CTLA-4 interactions.

### Native cell-expressed CTLA-4 is uniquely capable of releasing PD-L1, regardless of CD80 expression levels

**Figure 5:**
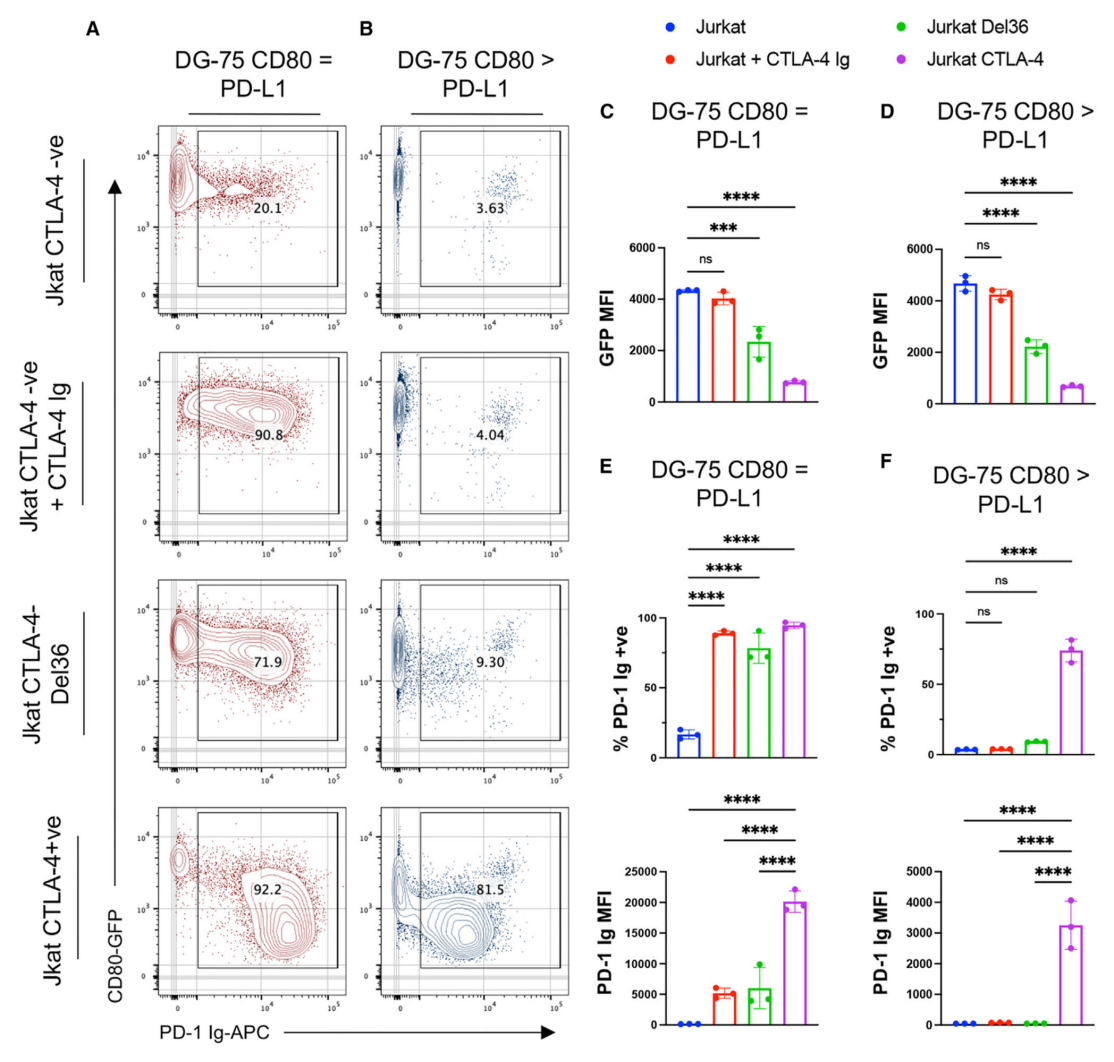
Only cell-expressed CTLA-4 restores PD-1 binding irrespective of CD80 and PD-L1 expression levels (A and B) Contour plots showing DG-75:CD80 = PD-L1 (A) and DG-75:CD80 > PD-L1 (B) incubated for 24 h with CTLA-4−ve Jurkat, CTLA-4−ve Jurkat +50 nM CTLA-4 Ig, CTLA-4-Del36 Jurkat, and CTLA-4+ve Jurkat. Cells were stained with 1 mg/mL of PD-1 Ig-APC after incubation. Data show representative fluorescence-activated cell sorting (FACS) plots of CD80-GFP vs. PD-1 Ig. (C–F) Graphical representations of (A) and (B) respectively, plotting CD80-GFP MFI, percentage of PD-1 Ig+ve cells and PD-1 Ig MFI on DG-75:CD80 = PD-L1 (C and E) and DG-75:CD80 > PD-L1 (D and F). Data are representative of three independent experiments showing mean ± SD. ***p ≤ 0.001, ****p ≤ 0.0001, ns, not significant: one-way ANOVA with Tukey’s multiple comparisons test (C–F).

Finally, we compared the ability of soluble CTLA-4 Ig proteins with cell-expressed WT CTLA-4 to release PD-L1. Here, we incubated DG-75 cells co-expressing CD80 and PD-L1 with either a WT CTLA-4+ve Jurkat T cell or a CTLA-4-negative Jurkat cell supplemented with 50 nM of CTLA-4 Ig, a dose sufficient to saturate CD80. Under equimolar conditions of PD-L1 and CD80 expression (Figure 5A), both CTLA-4 Ig and cell-expressed WT CTLA-4 were able to release PD-L1, as measured by PD-1 Ig binding. When comparing our generated CTLA-4 variants, only CTLA-4 C120A Ig was able to partially release PD-L1, although at much lower levels than WT CTLA-4 Ig (Figure S5). Interestingly, CTLA-4-Del36, a CTLA-4 mutant lacking the cytoplasmic tail essential for *trans*-endocytosis, also partially restored PD-1 binding but proved much less effective than CTLA-4 Ig or cell-expressed WT CTLA-4. In contrast, when using DG-75 cells expressing excess CD80, which is a more exacting scenario, only cell-expressed WT CTLA-4 was capable of restoring PD-1 Ig binding (Figure 5B). Moreover, cell-expressed WT CTLA-4 was able to deplete CD80 ligand by *trans*-endocytosis, as measured by loss of CD80-GFP from the DG-75 (Figures 5C and 5D).

In addition, although soluble CTLA-4 restored PD-1 Ig binding to the entire DG-75 population expressing equimolar levels of CD80 and PD-L1 (Figure 5E, top graph), incubation with WT CTLA-4+ve Jurkat cells resulted in increased PD-1 Ig binding, as measured by median fluorescence intensity (MFI) (Figure 5E, bottom graph). Here, we observed an almost 4-fold difference in PD-1 Ig MFI between CTLA-4 Ig-mediated release of PD-L1 and cell-expressed WT CTLA-4, suggesting that cell-bound CTLA-4 is more effective at releasing PD-L1 when compared with soluble CTLA-4 Ig and CTLA-4 Del36. These results highlight the importance of endocytic, cell-expressed CTLA-4, which is capable of both binding to CD80 and physically depleting the ligand. In contrast, CTLA-4 Ig and CTLA-4-Del36 are less able to release PD-L1 and are dependent on permissive levels of CD80 and PD-L1 expression (Figures 5E vs. 5F). Thus, only through *trans*-endocytosis of CD80 can CTLA-4 effectively release all PD-L1, regardless of the relative CD80 to PD-L1 expression levels.

## Discussion

The CTLA-4 and PD-1 pathways are major targets for therapeutic intervention in the treatment of autoimmunity, transplantation, and cancer. Recently, a significant molecular overlap between these pathways has been identified, mediated by the *cis* CD80/PD-L1 interaction. The impact of this interaction is to disable PD-L1 function and place it under the control of CD80 and CTLA-4 in some circumstances.

Several studies have now shown that by forming heterodimers with PD-L1, CD80 physically obstructs the PD-1 binding site, thereby acting as an antagonist to PD-1 signaling.^19,20^ The impact on CD28 and CTLA-4 appears much more subtle, consistent with the CD28/CTLA-4 binding site being on the opposite face of CD80 as compared with the PD-L1 binding interface. While one study suggested that CD80/PD-L1 interactions affected CTLA-4 function,^32^ these results contrast with several others where the impact on CTLA-4 binding appears intact.^21–23^

We previously demonstrated that cell-expressed CTLA-4 could both bind and *trans*-endocytose CD80, despite its interaction with PD-L1.^22^ Interestingly, depletion of CD80 by CTLA-4 demonstrated remarkable specificity, as removal of CD80 had no impact on PD-L1 levels, despite clear evidence of CD80/PD-L1 heterodimers on the membrane. Thus, in the context of CD80 and PD-L1 co-expression, CTLA-4 *trans*-endocytosis of CD80 releases competent PD-L1 at the membrane, enabling PD-1-mediated inhibition. Consistent with our findings, studies by Tekguc et al. likewise found that soluble CTLA-4 interactions and CTLA-4 trogocytosis could also liberate PD-L1.^23^ However, the mechanism by which CTLA-4 liberates free PD-L1 remains unresolved. Here, we have investigated the requirements for CTLA-4 to liberate PD-L1 from CD80/PD-L1 heterodimers. Our data reveal a mechanism whereby rigid, bivalent binding of CTLA-4 is required to orientate CD80 in the membrane such that PD-L1 binding is no longer favorable. However, soluble bivalent CTLA-4 molecules and CTLA-4 molecules incapable of *trans*-endocytosis only partially release PD-L1 at specific (approximately equimolar) ratios of CD80 to PD-L1 and fail under conditions of excess CD80 expression. In contrast, cell-expressed WT CTLA-4 molecules can utilize *trans*-endocytosis to continually deplete CD80, allowing effective PD-L1 release regardless of CD80/PD-L1 expression ratios.

Several lines of evidence support the above conclusions. A recent crystal structure of a high-affinity CD80 vIgD domain in complex with human PD-L1^30^ has provided new insights. Here, Maurer et al. showed that the CD80 binding interface for PD-L1 is situated along the ABED strands of the membrane-distal IgV domain, in line with previous mutational data.^20,21^CD80 homodimerization, which also uses this interface,^29^ is therefore incompatible with PD-L1 binding. In membranes, CD80 can exist dynamically as CD80 homodimers, heterodimers with PD-L1, or monomers due to the low-affinity interaction and non-covalent nature of both the homodimer and heterodimer.^18,29,33^ The structural data by Maurer et al. make clear that CTLA-4 binding to the opposite (GFCC′C″) face of the CD80 IgV domain is unimpeded by PD-L1. Thus, there appears to be no obvious structural incompatibility preventing CTLA-4 and PD-L1 from both binding to the same CD80 molecule and forming a tripartite complex. Moreover, there is no obvious reason why CTLA-4 binding to CD80 should physically displace PD-L1. This is compatible with our observations using soluble monovalent or flexible CTLA-4 molecules, which bind to CD80/PD-L1 heterodimers but do not trigger the release of PD-L1.

Alignment of the CD80/PD-L1 and WT CD80 structures indicates that the PD-L1/CD80 interaction occurs with PD-L1 adopting an unusual lying down position. In homodimers, CD80 molecules are essentially parallel to each other and orthogonal to the membrane. However, Maurer et al.^30^ commented that the orthogonal angle of CD80 in the membrane would be unfavorable for CD80/PD-L1 heterodimer formation. Accordingly, in the CD80/PD-L1 heterodimer, CD80 can no longer maintain its “upright” position, seen in the CD80 homodimer. Conversely, if CTLA-4 positions CD80 in an upright position, then this becomes unfavorable for PD-L1 binding. Controlling the angle of CD80 in the membrane therefore appears crucial to the ability of CTLA-4 to disrupt the PD-L1/CD80 heterodimer. Our observations suggest that bivalent CTLA-4 binding (e.g., cross-linking) of two CD80/PD-L1 heterodimers is precluded. However, due to the relatively weak affinity (∼20 μM) of CD80 for PD-L1,^18,33^ CD80 and PD-L1 will frequently associate and dissociate, whereupon the high-affinity bivalent binding of CTLA-4 to CD80 (0.2 μM) would force CD80 upright in the membrane, inhibiting its re-association with PD-L1. The strong bias toward CD80-CTLA-4 interactions explains why CTLA-4 binding releases PD-L1 from CD80, whereas PD-L1 has little impact on CTLA-4-CD80 interactions. Indeed, when we co-express CD80 with excess PD-L1 on cells (such that all CD80 is heterodimerized), we observe increased staining with CTLA-4 Ig, consistent with enhanced monovalent binding to CD80. This is predicted since twice as much CTLA-4 Ig can bind monovalently as bivalently. In contrast, the decreased staining seen by others^32^ may relate to different expression levels of CD80 and PD-L1 or concentrations of CTLA-4 Ig used.

Prevention of CD80 re-binding to PD-L1 appears to be dependent on the rigidity of CTLA-4. CTLA-4 is normally expressed as a covalent homodimer by virtue of a disulfide bond located at the base of the stalk region. The stalk region also contains reciprocal hydrophobic and hydrophilic interactions up to the A′ strand of both IgV domains.^31^ Such non-covalent interactions, supported by the disulfide bridge at the base of the homodimer, contribute rigidity to the fixed angle between the two IgV domains of the CTLA-4 homodimer. It is interesting to note that CD28 has a different dimer angle, which does not allow bivalent CD80 binding,^4,26^ consistent with its inability to release PD-L1. We observed that replacing the CTLA-4 stalk region with a flexible glycine-serine linker removed the ability of CTLA-4 to promote dissociation of the PD-L1/CD80 heterodimer. Thus, a flexible CTLA-4 dimer can bind bivalently to CD80/PD-L1 complexes without releasing PD-L1. Interestingly, when only the cysteine residue is mutated to an alanine, therefore retaining only the non-covalent interactions in the stalk region, CTLA-4 C120A Ig appeared to partially release PD-L1, although at much lower levels than WT CTLA-4 Ig. Thus, our data argue that both bivalent and rigid binding, associated with the natural CTLA-4 homodimer configuration, is required to orient CD80 in an upright fashion in the membrane, thereby precluding CD80/PD-L1 interactions.

We also observed that rigid-bivalent CTLA-4 Ig is only able to release PD-L1 when CD80 and PD-L1 are expressed at approximately equimolar ratios yet is incapable of disrupting the CD80/PD-L1 interaction when the CD80 ligand is in excess of PD-L1. Under conditions of excess CD80 expression, CD80 homodimers will be common, and CTLA-4 Ig will only bridge two heterodimers infrequently. We hypothesize that the flexibility of monomeric (and potentially homodimeric) CD80 is such that CTLA-4 bivalent binding between a CD80/PD-L1 heterodimer complex and CD80 is not precluded, thereby maintaining the CD80/PD-L1 interactions and not releasing PD-L1. In contrast, where cross-linking of two CD80/PD-L1 heterodimers is favored, CTLA-4 Ig promotes the release of PD-L1. Crucially, depletion of CD80 by CTLA-4 *trans*-endocytosis continually adjusts the ratio of CD80 to PD-L1 at the cell membrane. As CD80 is depleted, the relative frequency of CD80/PD-L1 heterodimers increases, eventually reaching ratios of both ligands, where CTLA-4 bivalency and rigidity contribute to the disruption of CD80/PD-L1 heterodimers. This ability to continually remove CD80 by cell-expressed CTLA-4 explains why WT CTLA-4 is capable of potentiating PD-L1-PD-1 interactions regardless of CD80 expression levels.

The above concepts are relevant to abatacept, a dimeric CTLA-4 Ig fusion protein that has been clinically approved for autoimmunity.^9–11,13^ One possibility is that abatacept could function to some extent via promoting PD-L1-PD-1 interactions. However, our data would suggest that this is unlikely for two reasons. First, only specific ligand expression levels are permissive for the disruption of CD80/PD-L1 interactions, meaning that this effect would be limited to certain APC populations. Second, the MFI of PD-1 Ig binding after treatment with CTLA-4 Ig suggests that CTLA-4 Ig may only partially release PD-L1, in contrast with cell-expressed WT CTLA-4, which can potentially liberate all PD-L1. Moreover, abatacept may also prevent cell-expressed CTLA-4 from interacting with CD80 and, thereby, inhibit the release of PD-L1 by *trans*-endocytosis. Another relevant consideration is that a secreted soluble CTLA-4 isoform might also affect PD-L1 activity.^34,35^However, this splice variant lacks the cysteine responsible for forming covalent dimers and displays changes in the stalk region, which appear to preclude both non-covalent and covalent interactions. Soluble CTLA-4 is therefore likely to be monomeric and unable to release PD-L1.

Taken together, our data provide an explanation for the specific removal of CD80 and release of PD-L1 by CTLA-4. Our observations indicate that structural characteristics, including the bivalency and rigidity of CTLA-4, alongside the angle of CD80 in complex with PD-L1 relative to the cell membrane, are essential for disruption of the CD80/PD-L1 heterodimer. Moreover, our results indicate that release of PD-L1 by soluble CTLA-4 is determined by levels of CD80 expression, whereby PD-L1 may only be released after sufficient depletion of CD80. Our previous studies indicated that CD80 *trans*-endocytosis can also drive CTLA-4 ubiquitination^6^; how CD80/PD-L1 heterodimers influence this aspect of CTLA-4 biology remains to be determined. Taken together, data herein show how CD80, PD-L1, and CTLA-4 act in concert to influence PD-1 biology, suggesting that CD80 can act as a master switch influencing the behavior of both the CTLA-4 and PD-1 pathways.

## Limitations of the study

In this study we have used model cell lines (Jurkat T cells and DG-75 B cells) to explore the interactions between PD-L1 and CD80. We show that distinct expression ratios of CD80 and PD-L1 impact the ability of soluble CTLA-4 molecules to release PD-L1. However, understanding how relative expression is regulated in immune settings and primary immune cells remains to be fully explored. Furthermore, in our model, we propose that the orientation of CD80 and PD-L1 proteins in the cell membrane accounts for PD-L1 release by CTLA-4. While this is consistent with data from crystallographic modeling and experiments with mutant CTLA-4 Ig proteins, direct evidence of such changes of the membrane orientation of proteins is lacking. The work uses soluble CTLA-4 Ig fusion proteins to assess the impacts of bivalency and rigidity on PD-L1 release. While this is relevant to how drugs such as abatacept may perform, further work is required to understand fully how this translates to the membrane CTLA-4 setting.

## Resource Availability

### Lead contact

Further information and requests for resources and reagents should be directed to and will be fulfilled by the lead contact, David M. Sansom (d. sansom@ucl.ac.uk).

### Materials availability

Requests for reagents may be directed to the corresponding authors.

## Star⋆ethods

### Key Resources Table

**Table:** 

REAGENT or RESOURCE	SOURCE	IDENTIFIER
Chemicals, peptides, and recombinant proteins		
RPMI 1640	Gibco	11875093
L-Glutamine	Gibco	25030081
Penicillin-Streptomycin	Gibco	15070063
Fetal Calf Serum (FCS)	Merck	F7524
Dulbecco’s Phosphate Buffer Saline, pH 7.4	Gibco	10010023
CD80-His	AcroBiosystem	B71-H5228
PD-1 Ig	Bio-Techne	1086-PD
CTLA-4 Ig (Abatacept)	MedChem Express	HY-108829
APC Lightning-Link Conjugation Kit	Abcam	ab201807
Dy405 Lightning-Link Conjugation Kit	Abcam	ab201798
Cell Trace Violet	ThermoFisher Scientific	C34557
Staphylococcal Enterotoxin E	Biomatik	RPC20109
Pierce™ TCEP-HCl	ThermoFisher Scientific	20490
QIAprep Spin Miniprep Kit	Qiagen	27104
Tween 20	Merck	9005-64-5
Bovine Serum Albumin	Merck	92100N
NEBuilder® HiFi DNA Assembly kit	New England Biolabs	E5520S
Experimental models: Cell lines		
Jurkat E6.1 T-cells	ATCC	TIB-152
DG-75 B-cells	ATCC	CRL-2625
Suspension CHOK1	Merck	85051005
Software and algorithms		
GraphPad Prism v9.02	GraphPad Software Inc.	N/A
FlowJo	BD Biosciences	N/A
Octet® Analysis Studio	Sartorius	N/A
ChimeraX	UCSF	N/A
Other		
HiTrap MabSelect SuRe Column	Cytiva	11003494
G2000SWXL HPLC Column	TSKgel	0008540
Anti-Penta-HIS (HIS1K) Biosensors	Sartorius	18-5120
Anti-human IgG Fc Capture (AHC) Biosensors	Sartorius	18-5060

## Experimental Model And Study Participant Details

Jurkat E6.1T cells and DG-75 B cells (ATCC) were cultured in RPMI 1640 (Gibco) supplemented with 10% FCS (Merck), 2 mM L-Glutamine (Gibco), 100 U/ml penicillin and 100 mg/mL streptomycin (Gibco). Cell lines were kept at 37°C, 5% CO_2_ in a humidified atmosphere.

Endogenous CD80 was KO with the CRISPR/Cas9 system from the DG-75, Burkitt’s’ B-cell lymphoma cell line. DG-75 cells were then transduced with CD80 and PD-L1 tagged at their C terminus with GFP and mCherry, respectively. Jurkat T-cells were transduced with WT CTLA-4 or CTLA-4 Del 36 cDNAs, the latter generated by insertion of a stop codon that interrupts transcription of the final 36 amino acids of the CTLA-4 cytosolic tail.

Cell line engineering was carried out as described previously.^22^

## Method Details

### Generation of CTLA-4 Ig fusion constructs

For this study, we generated three CTLA-4 hIgG1 Fc fusion constructs, with the peptide sequences of the CTLA-4 ectodomain specified in Figure S2. The CD28 Ig fusion protein included the full ectodomain of CD28 (N19-P152) fused to a hIgG1 Fc domain. The Fc domain used in these constructs contains a triple mutation (TM) which reduces Fc-mediated binding.^36^

To generate our constructs, DNA sequences of the various CTLA-4 and CD28 ectodomains and human Fc TM, knob Fc TM and hole Fc TM were codon-optimised for Chinese Hamster Ovarian (CHO) expression, ordered on GeneArt, and cloned into a proprietary mammalian expression vector with the NEBuilder HiFi DNA Assembly kit (NEB). Monovalent CTLA-4 constructs were generated using the knobs-into-holes technology,^28^ whereby the pairing of two distinct heavy chains is achieved by molecular complementarity of separate ‘knob’ and ‘hole’ heavy chains. Here, the sequence for CTLA-4 with the C120A mutation was inserted into a human gamma-1 constant heavy chain carrying the ‘knob’ mutation. The ‘knob’ heavy chain was paired with an empty ‘hole’ heavy chain, thus resulting in an Ig construct containing a single CTLA-4 monomer. Plasmid DNA was purified (QIAprep Spin Miniprep Kit, Qiagen), and successful cloning confirmed by Sanger sequencing (GENEWIZ, Azenta). The proteins were transiently expressed in CHO cells.^37^

6-day post-transfection cells were pelleted, and supernatant vacuum filtered. Cleared supernatant was loaded onto a MabSelect SuRe column (Cytiva) pre-equilibrated with DPBS (Dulbecco’s Phosphate Buffer Saline, Gibco) pH 7.4. Proteins were eluted with 0.1 M glycine pH 2.7 and immediately injected onto a size exclusion chromatography column (SEC, Superdex 200 Increase HiScale 26/40, Cytiva) equilibrated and ran in DPBS pH 7.4. Purity and chain composition were assessed by analytical size exclusion chromatography, with a purity threshold >85% of monomeric protein (Agilent HPLC (1260 Infinity II LC System), TSKgel G2000SWXL HPLC Column, phase diol, L × I.D. 30 cm × 7.8 mm, 5 μm particle size).

### BLI analysis

The affinity of CTLA-4 and CD28 constructs for CD80 was measured by BLI with the OctetRED384 (Sartorius). All samples were prepared in BLI buffer (DPBS supplemented with 0.02% Tween 20 and 0.1% bovine serum albumin (Merck)). Serial dilutions of Human CD80, His Tag (CD80-His, AcroBiosystem) were made as indicated in figure legends.

Biosensors were equilibrated in BLI buffer for 10 min before start of assay. Baseline measurements were taken before loading of CTLA-4 or CD28 construct on Anti-human IgG Fc Capture (AHC) Biosensors (Sartorius). After reaching a displacement of 0.8–1 nm, biosensors were transferred into BLI buffer for a second baseline. For association step, biosensors were moved into wells containing serial dilution of CD80-His, and finally transferred into wells containing BLI buffer for dissociation step. Kinetic results were fit to a 1:1 model using global fitting, with y axis alignment on the average of a segment of the dissociation step, using the Octet Analysis Studio Software.

Avidity measurements were performed as above, with CD80-His immobilised on Anti-Penta-HIS (HIS1K) Biosensors (Sartorius), and association occurring with serial dilution of CTLA-4 constructs, as indicated in figure legend.

### CTLA-4 and PD-1 cell binding assays

PD-1 Ig (Bio-Techne), CTLA-4 Ig (Abatacept, MedChem) and in-house generated CTLA-4 and CD28 Ig constructs were conjugated with APC or Dy405 using the Lightning-Link Conjugation kits (Abcam), according to manufacturer’s instructions. 2 x 10^5^ DG-75 cells were incubated with indicated concentration of conjugated CTLA-4 or CD28 constructs for 30 min at 37°C. Cells were washed once in PBS followed by 15 min stain with 1 μg/mL of PD-1 Ig – Dy405, at 37°C. Cells were washed twice in PBS followed by fixation in 4% PFA.

For *trans*-endocytosis assays, DG-75 cells were first stained with Cell Trace Violet according to manufacturer’s instructions. 1 x 10^5^ DG-75 cells were co-cultured with 1 x 10^5^ Jurkat T-cells expressing either WT CTLA-4, CTLA-4-Del36 or no CTLA-4. Wells were supplemented with 5 ng/mL of Staphylococcal Enterotoxin E (BioMatik) to enhance cell-cell contacts. Assay was carried out in round-bottom, 96-well plate, in a total volume of 200 μL, for 24 h at 37°C. Cells were then washed once in PBS followed by 15 min stain with 1 μg/mL of PD-1 Ig – APC, at 37°C. Cells were washed twice in PBS followed by fixation in 4% PFA. In all cases, cells were resuspended in FACs buffer and acquired on the BD Fortessa.

### Quantification And Statistical Analysis

Statistical analyses and significance were determined using GraphPad Prism v9.02 software (GraphPad Software Inc.). Experiments were performed in biological triplicate and statistical significance determined with appropriate test, as indicated in figures.

Flow cytometry data was post-processed with FlowJo (BD Biosciences). Structural models of proteins were accessed from PDB and analyzed with UCSF ChimeraX. Alignment was performed using the matchmaker tool.

## Supplementary Material

Supplemental information can be found online at https://doi.org/10.1016/j.celrep.2024.114768.

Supplementary Materials

## Data Availability

Data may be made available upon request from the lead contact. This paper does not report original code. Any additional information required to reanalyze the data reported in this work paper is available from the lead contact upon reasonable request.

## In brief

The *cis* interaction between CD80 and PD-L1 disrupts PD-L1 function. Robinson et al. show that CTLA-4 restores PD-L1 activity, but this requires rigid bivalent binding of CTLA-4 to CD80. The unusual angle of the PD-L1-CD80 interaction indicates that PD-L1 release occurs via a physical reorientation of CD80, which is then unfavorable for PD-L1 binding.

## Highlights

- *Cis* PD-L1 binding re-orientates CD80 in the membrane
- Release of PD-L1 from CD80 requires rigid bivalent binding by CTLA-4
- Soluble CTLA-4 only releases at specific PD-L1:CD80 expression ratios
- CTLA-4 *trans*-endocytosis of CD80 efficiently releases PD-L1
