# Supplementary material for: Rigid, bivalent CTLA-4 binding to CD80 is required to disrupt the cis CD80/PD-L1 interaction
Source: Cell Rep. Author manuscript; Available in PMC 2026 Feb 19. (PMC7618755; doi:10.1016/j.celrep.2024.114768)
Supplement: Supplementary Materials [file EMS212457-supplement-Supplementary_Materials.pdf]

Cell Reports, Volume *43*

Supplemental information

Rigid, bivalent CTLA-4 binding to CD80 is required to disrupt the *cis* CD80/PD-L1 interaction

Maximillian A. Robinson, Alan Kennedy, Carolina T. Orozco, Hung-Chang Chen, Erin Waters, Dalisay Giovacchini, Kay Yeung, Lily Filer, Claudia Hinze, Christopher Lloyd, Simon J. Dovedi, and David M. Sansom

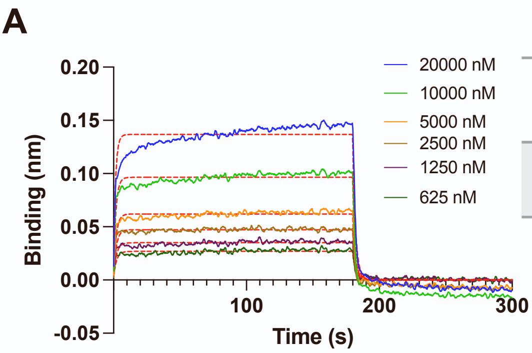

	K_D (M)	k_{on} (M^{-1} s^{-1})	k_{off} (s^{-1})
**CD28 Ig**	(4.27 ± 0.46) x 10^{-5}	(1.06 ± 0.1) x 10^{4}	(4.51 ± 0.08) x 10^{-1}

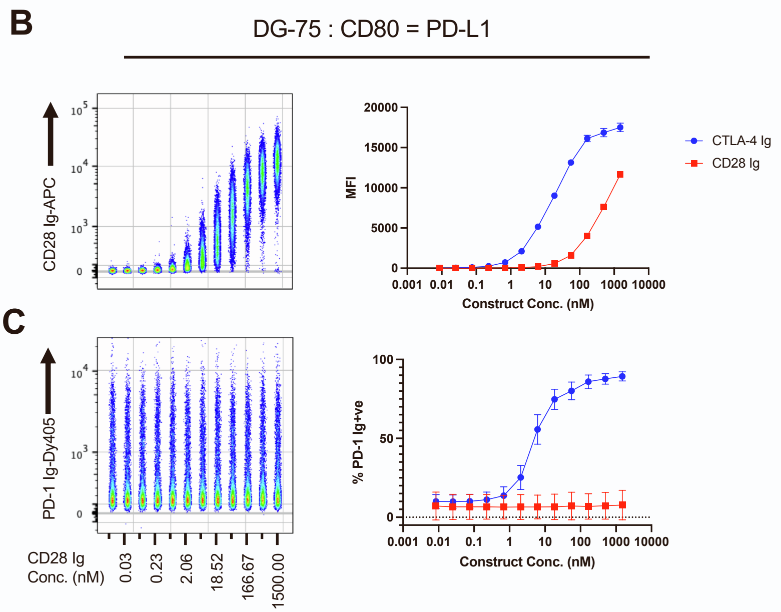

Fig S1. Soluble CD28 Ig fails to disrupt the CD80 PD-L1 interaction (related to Fig.1)
(A) Binding curves showing the association and dissociation of 20, 10, 5, 2.5, 1.25, 0.625 uM of CD80-His to CD28 Ig. Red lines show best fit to a 1:1 binding model. Right hand table details kinetic and thermodynamic parameters (K_D, k_{on}, k_{off}) obtained from the best global fit of the association/dissociation data to a 1:1 binding model. The errors given are fitting errors from the global fitting. **(B)** Concatenated flow cytometry plot of a 12-point serial dilution of CD28 Ig-APC, starting at 1500nM, on DG-75 : CD80 = PD-L1, with graphical representation in right-hand panel. **(C)** PD-1 Ig binding on cells described in (B), with graphical representation in right-hand panel. Data are representative of three independent experiments showing mean ± SD.

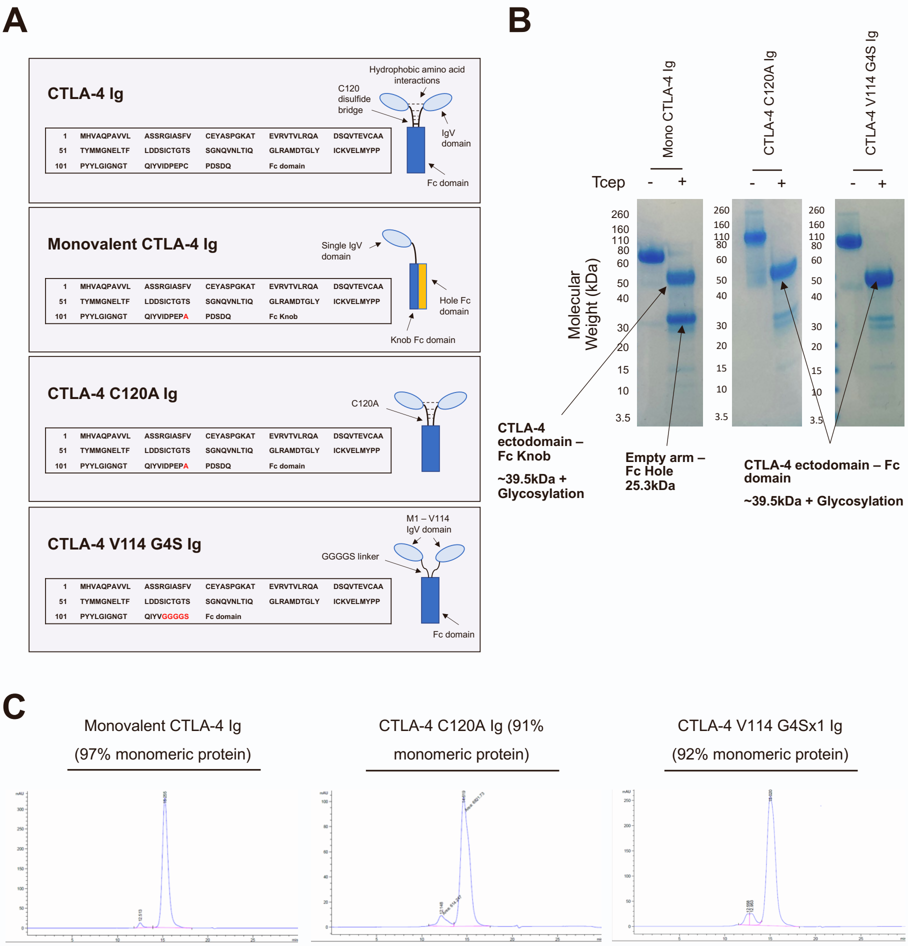

Fig S2. Design and quality control of novel CTLA-4 Ig constructs (related to Fig.2) **(A)** Amino acid sequences and schematics of CTLA-4 Ig constructs. **(B)** Gel electrophoresis of indicated CTLA-4 Ig construct, with annotated bands, +/- reducing agent Tcep. **(C)** Size exclusion traces of purified CTLA-4 Ig proteins, with indicated percentage of monomeric construct.

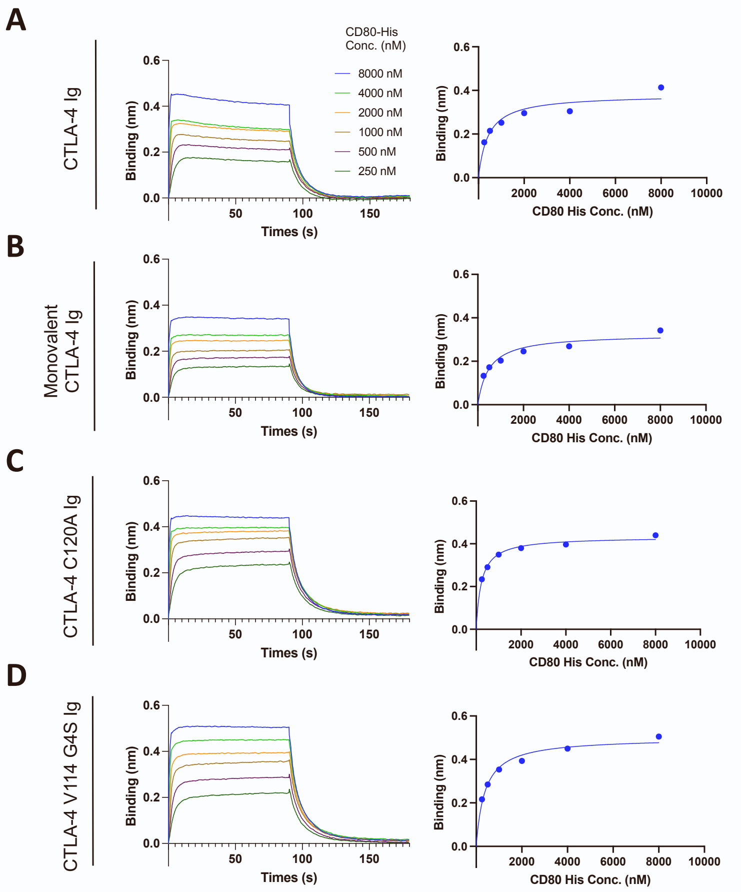

E

	K_D (M)	R_{max} (nm)
**CTLA-4 Ig**	(4.2 ± 2.55) x 10^{-7}	(0.380 ± 0.05)
**Monovalent CTLA-4 Ig**	(4.79 ± 2.40) x 10^{-7}	(0.325 ± 0.04)
**CTLA-4 C120A Ig**	(2.31 ± 0.4) x 10^{-7}	(0.433 ± 0.01)
**CTLA-4 V114 G4S Ig**	(3.8 ± 0.93) x 10^{-7}	(0.500 ± 0.03)

Fig S3. Steady-state analysis of CTLA-4 constructs (related to Figure 2)
(A-D) Binding curves showing association and dissociation of 8000, 4000, 2000, 1000, 500, 250 nM of CD80-His to CTLA-4 WT Ig (A), Monovalent CTLA-4 Ig (B), CTLA-4 C120A Ig (C) and CTLA-4 V114 G4S Ig (D). Right-hand graphs show the nonlinear curve fitting to the binding response at equilibrium (averaged between 50-80 secs) of CD80 His to indicated construct. **(E)** Steady-state analysis of CD80-His binding to indicated construct, including dissociation constant (K_D) and binding maximum (R_{max}), obtained from the one site – specific binding model with least-squares fit.

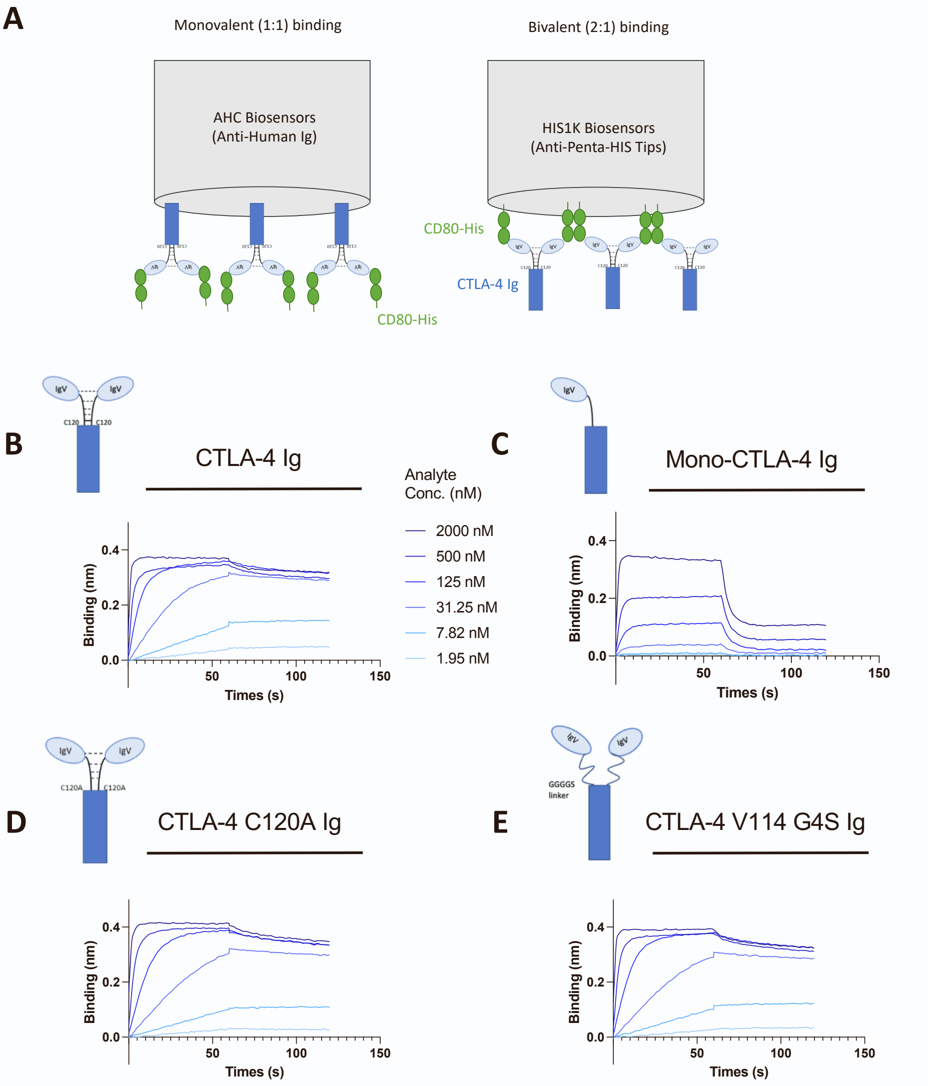

Fig S4. Bivalent CTLA-4 constructs show evidence of enhanced avidity towards CD80 (related to Figure 2).

(A) Schematic of Bio-Layer Interferometry assays assessing 1:1 and 2:1 binding models. **(B-D)** Binding curves showing the association and dissociation of 2000, 500, 125, 31.25, 7.82 and 1.95 nM of indicated CTLA-4 Ig constructs to CD80-His.

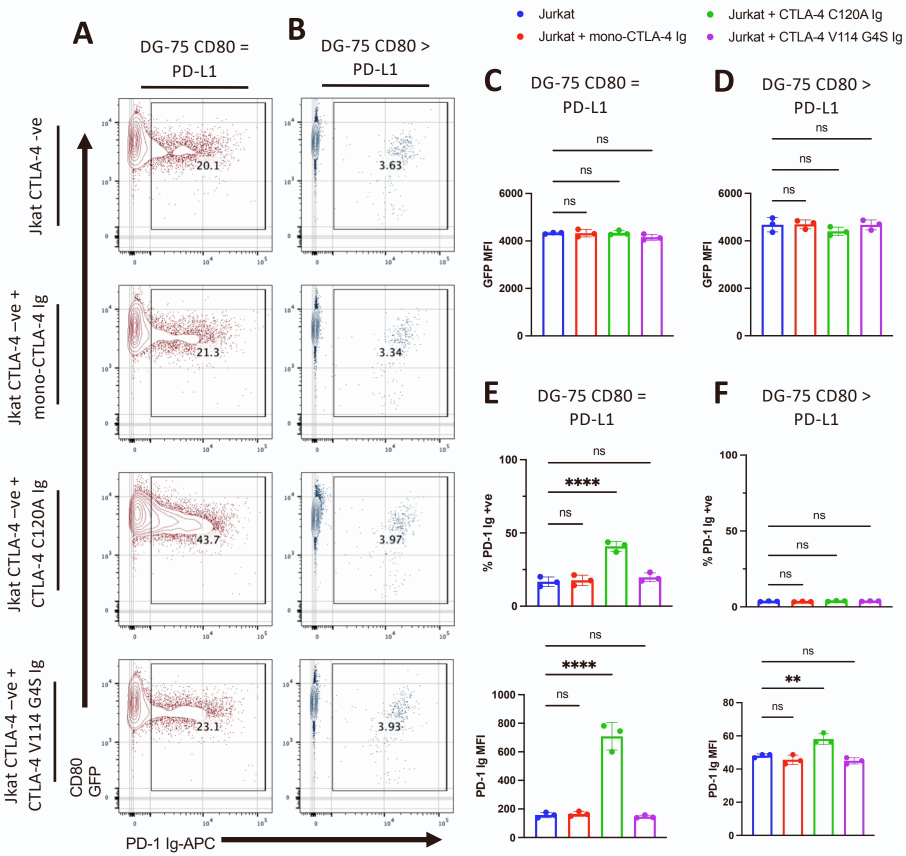

Fig S5. Monovalent and flexible soluble CTLA-4 constructs fail to restore PD-1 binding (related to Figure 5).

(A & B) DG-75 : CD80 = PD-L1 (A) and DG-75 : CD80 > PD-L1 (B) incubated for 24hrs with either: CTLA-4-ve Jurkat, CTLA-4-ve Jurkat + 50nM mono-CTLA-4 Ig, CTLA-4-ve Jurkat + 50nM CTLA-4 V114 G4S Ig and CTLA-4-ve Jurkat + 50nM CTLA-4 C120A Ig. Cells were stained with 1ug/ml of PD-1 Ig-APC after incubation. Data shows representative FACS plots of CD80-GFP vs. PD-1 Ig. **(C-F)** Graphical representation of (A & B) respectively, plotting CD80-GFP MFI, % of PD-1 Ig +ve cells and PD-1 Ig MFI on DG-75 : CD80 = PD-L1 (C,E) and DG-75 : CD80 > PD-L1 (D,F). Data are representative of three independent experiments showing mean ± SD. **P ≤ 0.01, ****P ≤ 0.0001, ns, not significant: one-way ANOVA with Tukey's multiple comparisons test (C-F).
